# Mitral valve prosthesis endocarditis unveiling metastatic colorectal cancer as the primary infection source: a case study

**DOI:** 10.1186/s43044-026-00718-7

**Published:** 2026-02-02

**Authors:** Abdellah Boucetta, Obeida Saleh, Badr Abdallani, Meryem Haboub, Abdenasser Drighil

**Affiliations:** Centre Hospitalier Universitaire Ibn Rochd, Casablanca, Morocco

## Abstract

**Background:**

This case report is significant due to its illustration of how infectious endocarditis on a prosthetic mitral valve revealed an underlying metastatic colorectal cancer. Although uncommon, associations between cardiac infection and advanced malignancy, particularly with malignancy serving as the source of infection, have been documented in the literature, making this case clinically significant and complex.

**Case presentation:**

A 54-year-old postmenopausal woman with insulin-dependent diabetes and poorly managed hypertension, who had previously undergone mitral valve replacement with a mechanical prosthesis and tricuspid valve repair for rheumatic mitral stenosis, was admitted for atrial fibrillation with rapid ventricular response. On admission, she presented with normochromic normocytic anaemia (7.7 g/dL), elevated inflammatory markers (CRP 250 mg/L), and leukocytosis (14,000/µL, neutrophils 10,000/µL). Transthoracic and transesophageal echocardiography identified vegetation on the mitral prosthesis with elevated gradients. Blood cultures were positive for Escherichia coli. A thoraco-abdominopelvic CT scan revealed a rectal tumor, confirmed by FDG-PET, with features consistent with metastatic colorectal cancer, considered the entry point for the infectious endocarditis. Tumor markers including ACE, CA 19 − 9, and CA 72 − 4 were elevated. The patient was treated with dual antibiotic therapy and showed initial clinical improvement but later died due to ventricular tachycardia.

**Conclusions:**

This case highlights the importance of considering atypical sources, such as metastatic cancer, in unresolved cases of endocarditis. It underscores the need for comprehensive diagnostic assessment, including oncologic evaluation, in patients with prosthetic valve endocarditis caused by atypical pathogens like E. coli. It also stresses the importance of multidisciplinary collaboration between cardiology and oncology teams. This case report is significant due to its illustration of how infectious endocarditis on a prosthetic mitral valve revealed an underlying metastatic colorectal cancer (CRC).

**Supplementary Information:**

The online version contains supplementary material available at 10.1186/s43044-026-00718-7.

## Background

Endocarditis on a prosthetic valve represents a severe complication of cardiac valve replacement surgery, with high morbidity and mortality. This case demonstrates how prosthetic valve endocarditis can be associated with metastatic colorectal cancer (CRC) as the primary infection site, emphasizing the intricate interplay between malignancy and cardiac infection. A multidisciplinary approach, coupled with comprehensive investigations, is essential for diagnosis and management. This includes echocardiography, microbiological cultures, tumor markers, imaging studies (CT, FDG-PET), and PCR/ELISA tests when necessary.

The patient’s death from ventricular tachycardia illustrates the severity of complications that can arise when metastatic cancers are involved. This case report contributes to the medical literature by providing a concrete example of how cardiac pathology may be linked to underlying malignancy, offering insights for clinicians on diagnosis and treatment.

## Case presentation

This case study presents a 54-year-old postmenopausal woman with insulin-dependent diabetes and hypertension. She had undergone mitral valve replacement with a mechanical prosthesis and tricuspid valve repair for severe rheumatic mitral stenosis.

**Fig. 1:**
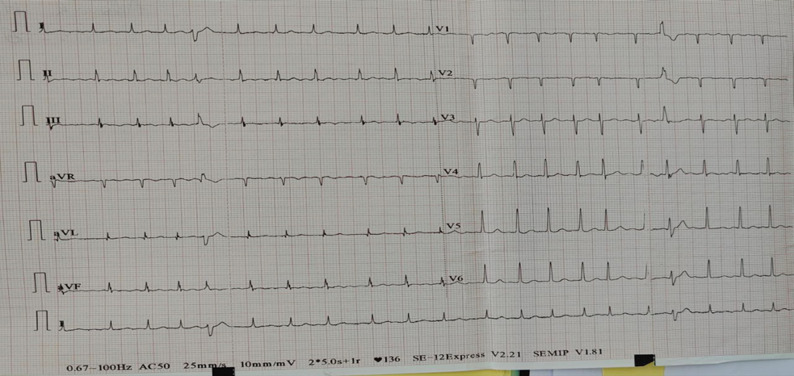
The electrocardiogram (ECG) demonstrates atrial tachycardia with ventricular extrasystoles

The patient presented with atrial fibrillation (AF) at a rate of 139 bpm with rapid ventricular response (Fig. 1), accompanied by a subjective sensation of fever but without measurable temperature. Investigations revealed normochromic normocytic anaemia (Hb 7.7 g/dL), elevated CRP (250 mg/L), and leukocytosis (14,000/µL, neutrophils 10,000/µL).

After transfusion of one unit of red blood cells, hemoglobin increased to 9.7 g/dL and heart rate control was achieved. The patient reported recent moderate rectal bleeding. Digital rectal examination revealed traces of blood without pain.

**Fig. 2:**
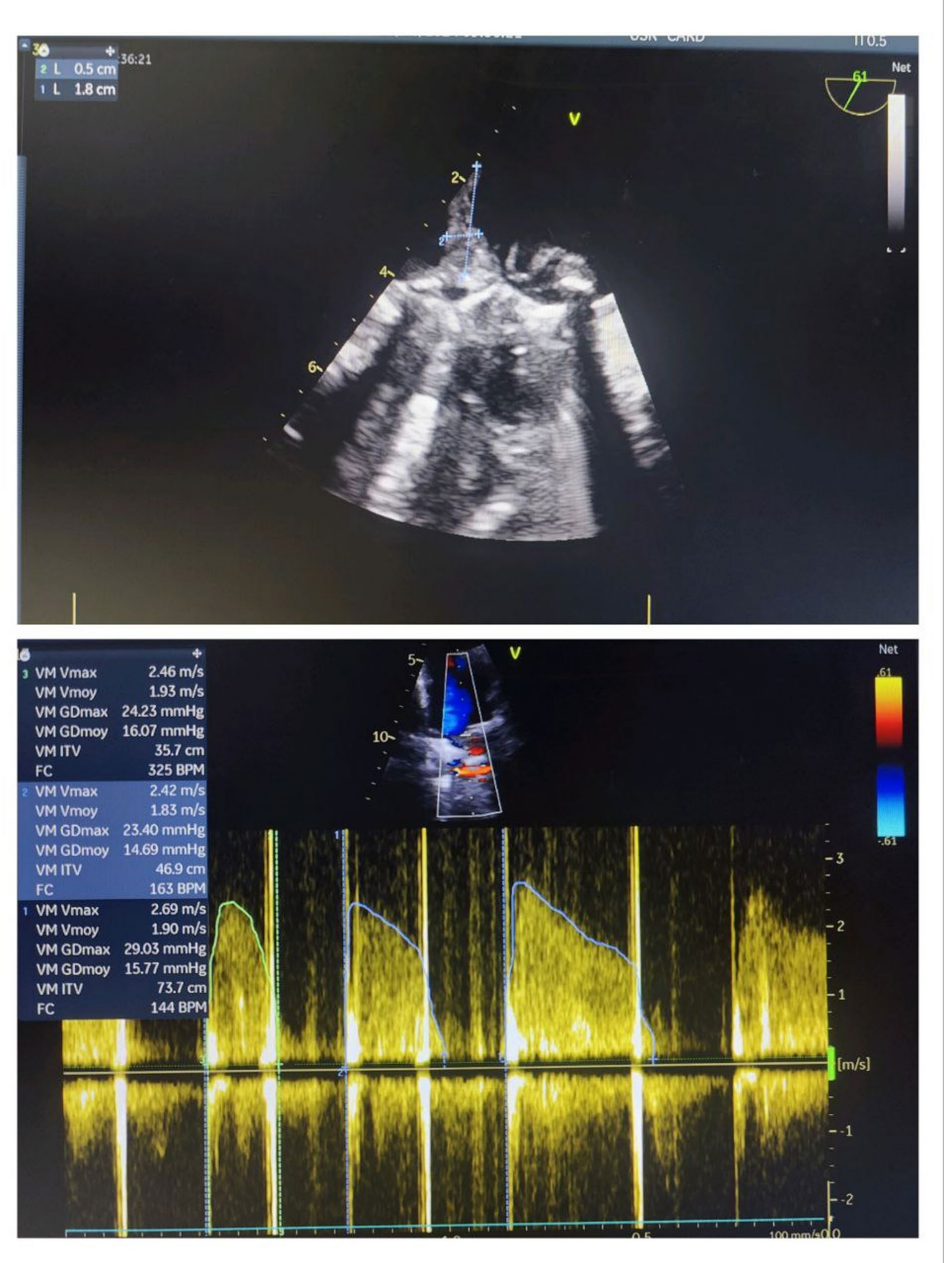
Transesophageal echocardiography (TEE), mid-esophageal mitral valve long-axis view at 61°, demonstrating a vegetation attached to the atrial aspect of the posterior leaflet of the mitral prosthesis, measuring 18 mm by 5 mm, associated with an elevated mean transprosthetic gradient of 15 mmHg

Transesophageal echocardiography (TEE) identified a vegetation on the atrial aspect of the posterior leaflet of the mitral prosthesis, measuring 18 × 5 mm, with elevated mean transprosthetic gradient (15 mmHg) (Fig. 2).

An urgent series of blood cultures (3 aerobic and 3 anaerobic with a one-hour interval) were positive for Escherichia coli. Consequently, a synergistic dual antibiotic therapy was initiated, tailored to the culture results: gentamicin 3 mg/kg/day and ceftriaxone 2 g.

An etiological assessment was conducted to identify the entry point; given the hemorrhagic syndrome, the thoraco-abdomino-pelvic CT scan identified a rectal tumor measuring approximately 4.5 cm, classified as cT3bN1Mx, with features suggestive of local invasion but no distant organ metastases beyond bone involvement. The FDG-PET scan further characterized the tumor’s metabolic activity with a maximal standardized uptake value (SUVmax) of 12.3, confirming its high metabolic activity consistent with malignancy, as well as a lytic lesion in the right iliac wing, bilateral pleural effusion, and nonspecific pulmonary micronodules.

These findings led to the diagnosis of probable metastatic colorectal cancer, considered as the entry point for infectious endocarditis on the mitral prosthesis with Escherichia coli.

**Fig. 3:**
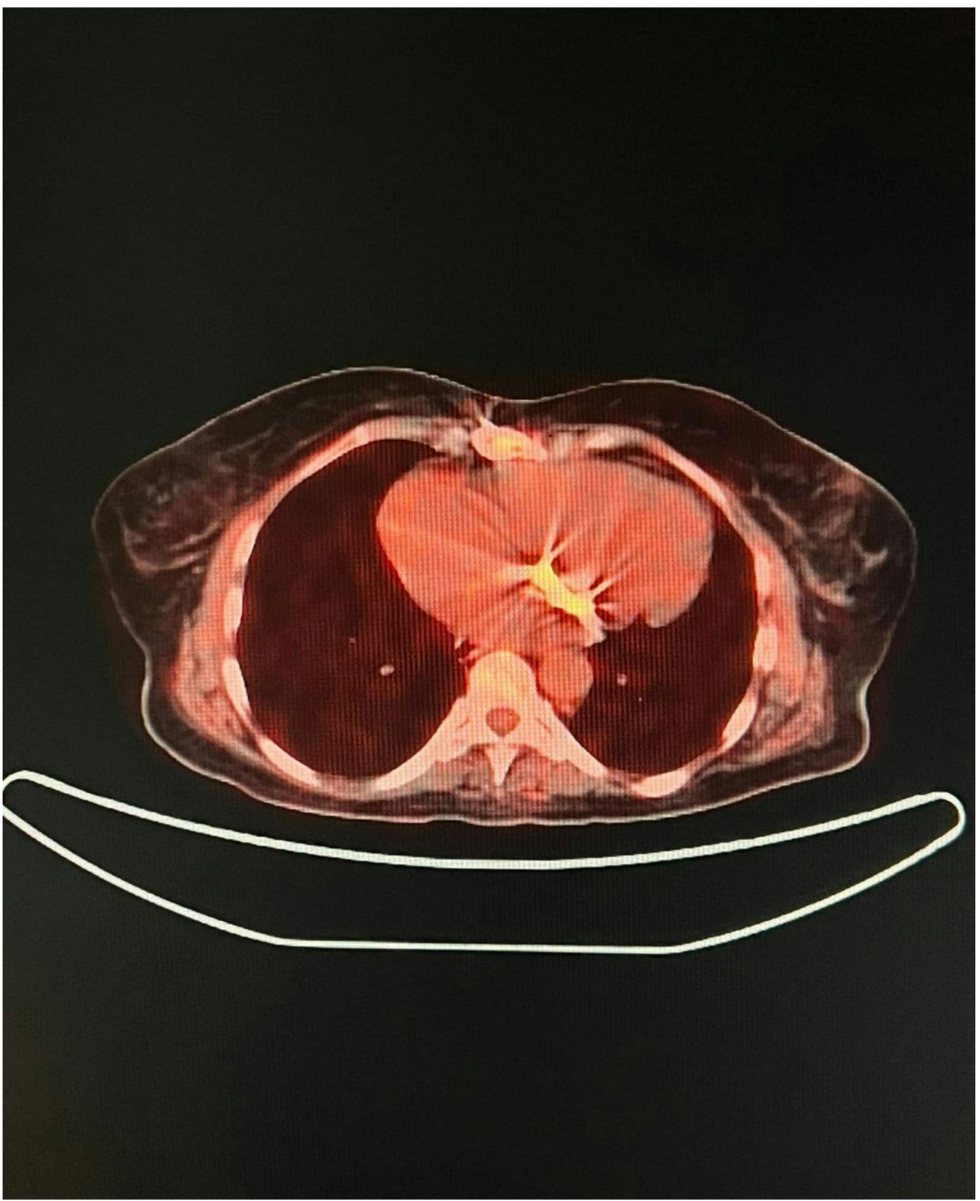
Positron emission tomography (PET) scan demonstrating pathological fluorodeoxyglucose (FDG) uptake at the site of the mitral valve prosthesis, consistent with active inflammatory or infectious process

**Fig. 4:**
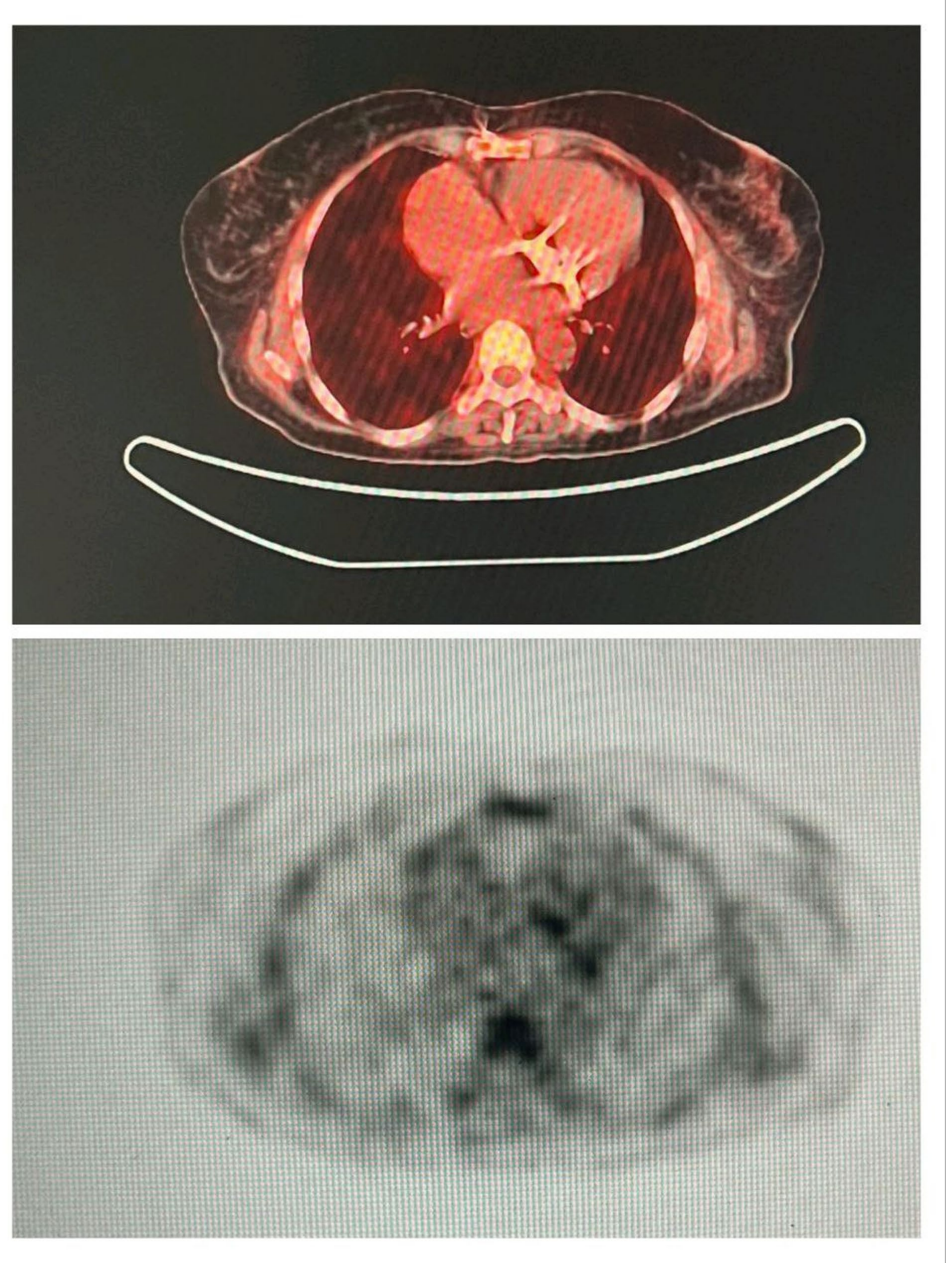
FDG-PET scan demonstrating pathological uptake in bone, consistent with metastatic involvement

Clinical evaluation was complemented by a fluorodeoxyglucose positron emission tomography (FDG-PET) scan, performed for a more detailed assessment of the malignancy and the mitral prosthesis. The FDG-PET imaging revealed pathological FDG uptake at the mitral prosthesis consistent with vegetation-related inflammation (Fig. 3), as well as abnormal FDG uptake in the bone suggestive of metastatic involvement (Fig. 4).

**Fig. 5:**
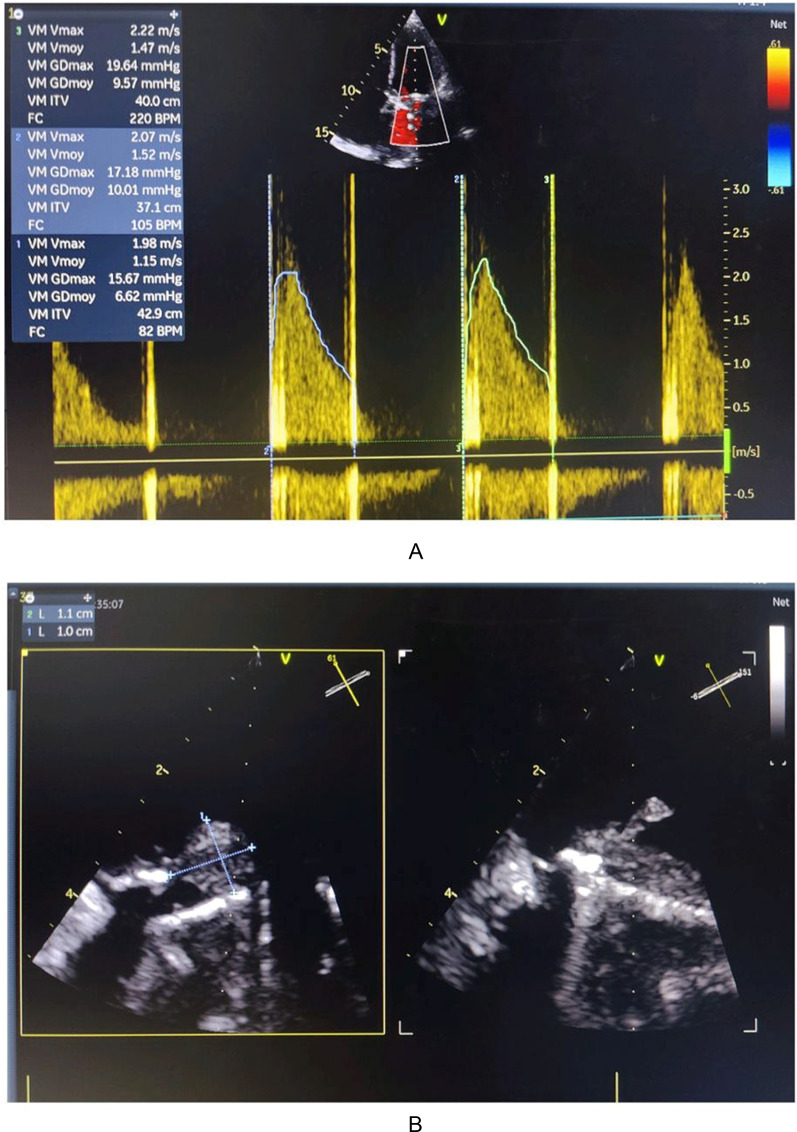
Transesophageal echocardiography images of the mitral prosthesis in mid-esophageal views at 150° (A) and 61° (B), demonstrating a reduction in vegetation size to 11 mm by 10 mm. There is resolution of the posterior leaflet obstruction and a decrease in the mean transprosthetic gradient to 10 mmHg

Cardiac progression was favorable under antibiotic therapy, with follow-up echocardiography showing a reduction in vegetation size to 11 mm by 10 mm with resolution of posterior leaflet blockage and a decrease in the gradient to 10 mmHg (Fig. 5).

**Fig. 6:**
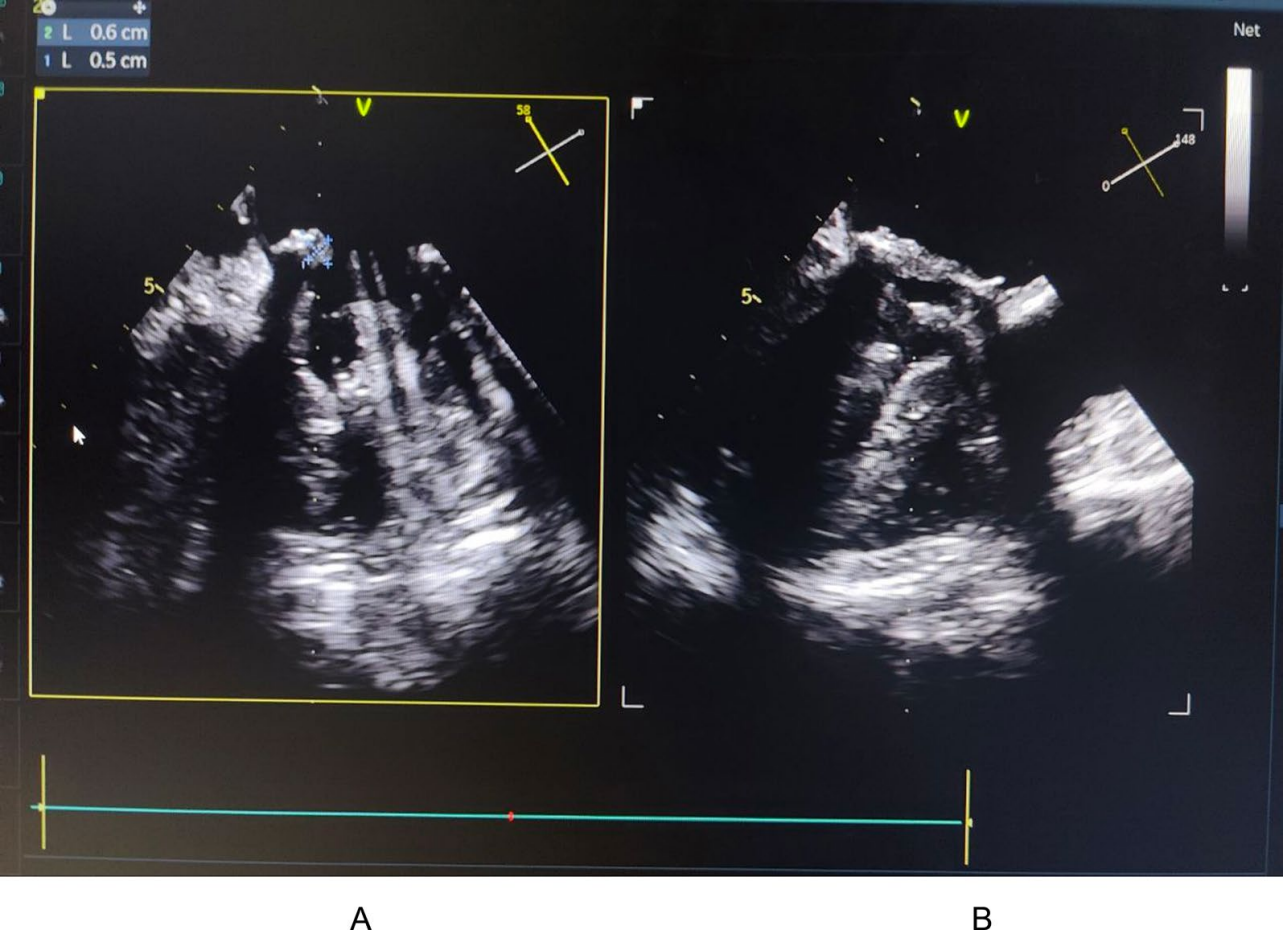
Transesophageal echocardiography images of the mitral prosthesis in mid-esophageal views at 148° (A) and 58° (B), showing a reduction in vegetation size to 5 mm by 6 mm, with resolution of posterior leaflet obstruction and a decrease in the mean transprosthetic gradient to 10 mmHg

C-reactive protein (CRP) levels significantly decreased from 250 mg/L to 100 mg/L in response to antibiotic treatment. Hemoglobin increased after the second transfusion, reaching 11 g/dL. After one month of treatment, there was a marked reduction in vegetation size to 5 mm by 6 mm with resolution of posterior leaflet blockage and a gradient decrease to 8 mmHg (Fig. 6).

**Fig. 7:**
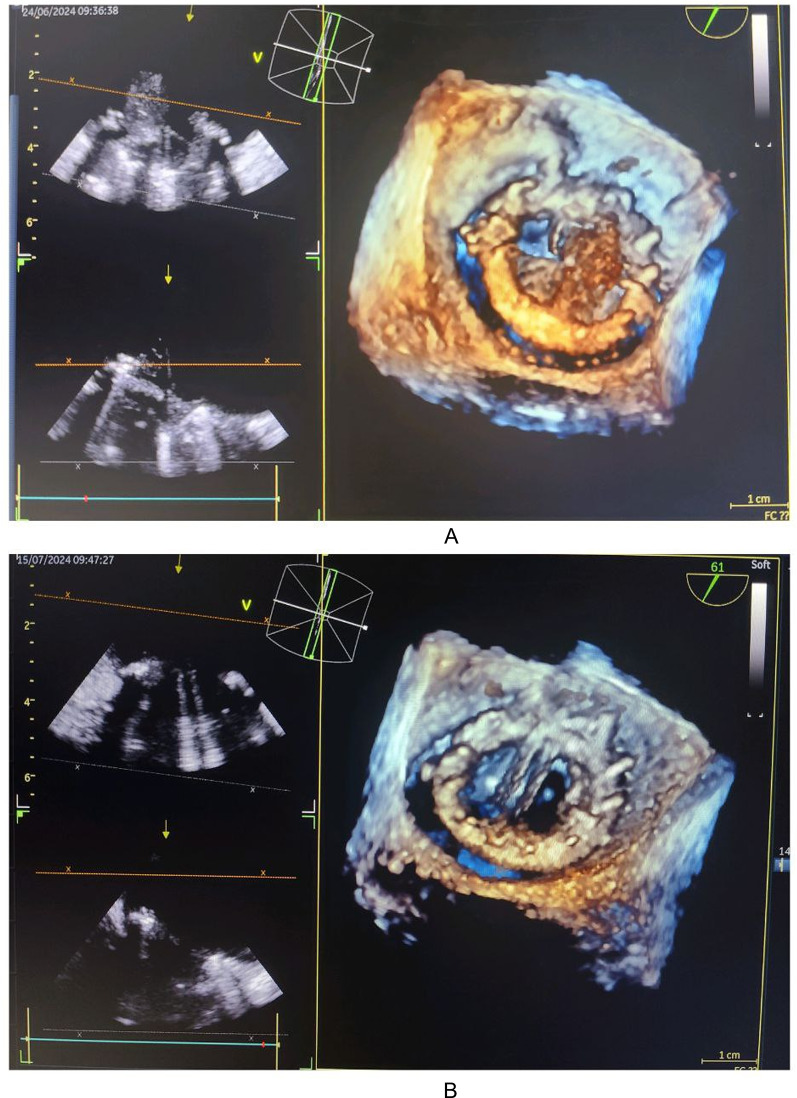
Trans-esophageal 3D echocardiography views at 45° (A) and 61° (B) before and after treatment, demonstrating significant regression of the vegetation and resolution of posterior leaflet obstruction

A trans-esophageal “3D” echocardiography before and after treatment showed a significant regression of the vegetation with leaflet release (Fig. 7).

Given this favorable evolution, the case was discussed with oncologists to eradicate the colorectal cancer considered as the entry point. A treatment plan including radiotherapy was proposed to treat the metastatic colorectal cancer. However, the patient unfortunately died due to ventricular tachycardia before this treatment could be implemented.

## Discussion

Infective endocarditis is a severe infection affecting the endocardium, particularly the cardiac valves. The increasing use of prosthetic valves has altered the epidemiological landscape of endocarditis, increasing the prevalence of infections on implanted material [1].

Prosthetic valve endocarditis (PVE) accounts for ~ 20% of all IE cases and has a worse prognosis than native valve IE [2].

E. coli, although rare in endocarditis (0.5–2% of cases), poses a particular risk in prosthetic valves due to biofilm formation. Infection can disseminate, causing septic emboli and systemic complications.

Prosthetic valve endocarditis has become a feared complication, representing about 20% of all cases of infective endocarditis (IE). Patients with prosthetic valve endocarditis have a darker prognosis compared to those with native valve endocarditis, due to the invasive nature of the infection and associated therapeutic challenges.

E. coli, a Gram-negative bacillus commonly found in the human gastrointestinal tract, is an unusual pathogen in endocarditis, but its implications for prosthetic valves, particularly the mitral valve, require special attention. Endocarditis caused by E. coli represents about 0.5% to 2% of cases of infective endocarditis, according to recent studies [3].

The occurrence of this infection on a mitral prosthesis is even rarer, representing a fraction of reported E. coli endocarditis cases.

In the registry of the prospective cohort study by the International Collaboration on Endocarditis, which included patients from 61 collaborative international hospitals, E. coli was the most common etiology among non-HACEK gram-negative rods (GNR) [4].

The registry included 2,761 patients with a definitive diagnosis of infective endocarditis (IE); 49 (1.8%) were caused by non-HACEK GNR [5]. E. coli accounted for only 14 (29%) cases in this group.

The rarity of this condition makes it difficult to accurately estimate its incidence, but it appears to be more frequent in patients with a history of urological or gastrointestinal manipulation, as in our case. One major impact of E. coli in this condition is its ability to form biofilms on the surface of the mitral prosthesis. These biofilms protect the bacteria from the host’s immune defenses and antibiotic treatments, making the infection difficult to eradicate and increasing the risk of recurrence [6].

Furthermore, the infection can disseminate, causing systemic complications such as septic emboli that can affect various organs, thus increasing the severity of the condition. The diagnosis of E. coli endocarditis on a mitral prosthesis is often delayed due to the subtle clinical presentation and the difficulty of culturing E. coli from biofilms. This delay can exacerbate the disease progression, making treatment more complex [7].

Cancers, particularly those associated with advanced neoplasms or paraneoplastic syndromes, can serve as entry points for infective endocarditis on a prosthetic valve [8]. The link between cancer and infective endocarditis can be explained by several immunopathological and clinical mechanisms: the combination of immunosuppression, frequent bacteremia, and a prothrombotic state creates a fertile ground for the development of infective endocarditis on prostheses [9].

In summary, colorectal cancer can not only facilitate E. coli entry into the bloodstream but also create conditions conducive to the establishment of infective endocarditis, particularly on a prosthetic valve. This link underscores the importance of close monitoring and proactive management of patients with CRC, especially those with predisposing factors like our case [10].

This case highlights the complexity of treating patients with prosthetic valve endocarditis associated with metastatic malignancy. Early identification of colorectal cancer as the source of the infection guided antibiotic treatment. However, the presence of an aggressive neoplasm significantly influenced the overall prognosis. Multidisciplinary management is crucial to optimize outcomes in these patients.

## Conclusion

This clinical case illustrates the necessity of a comprehensive diagnostic approach in prosthetic valve endocarditis, particularly in the absence of an evident source of infection.

The identification of metastatic colorectal cancer as the source of the infection is a rare but pivotal step in guiding therapeutic management. The concurrent management of the infection and prompt oncological treatment is essential for improving survival rates. However, in this case, the patient ultimately succumbed to a cardiac complication.

In conclusion, this article offers invaluable insights for healthcare professionals, providing a practical illustration of how prosthetic valve endocarditis can reveal metastatic cancers, underscoring the diagnostic and therapeutic challenges associated with these complex conditions.

## Supplementary Information

Below is the link to the electronic supplementary material.

Supplementary Material 1.

Supplementary Material 2.

Supplementary Material 3.

## Data Availability

No datasets were generated or analysed during the current study.

## Abbreviations

AF: Atrial fibrillation (Fibrillation auriculaire)
bpm: Beats per minute (Battements par minute)
CRP: C-reactive protein (Protéine C-réactive)
CT TAP: Computed tomography thoraco-abdomino-pelvienne
ECG: Electrocardiogram
E. coli: Escherichia coli
PET: Positron emission tomography (Tomographie par émission de positrons)
FDG-PET: Fluorodeoxyglucose positron emission tomography
IE: Infective endocarditis (Endocardite infectieuse)
CRC: Metastatic colorectal cancer
ExPEC: Extraintestinal pathogenic Escherichia coli
