# Supplementary material for: Mitral valve prosthesis endocarditis unveiling metastatic colorectal cancer as the primary infection source: a case study
Source: Egypt Heart J. 2026 Feb 2;78:8. doi: 10.1186/s43044-026-00718-7 (PMC12864643; doi:10.1186/s43044-026-00718-7)
Supplement: Supplementary file 1 — Supplementary Material 1. [file 43044_2026_718_MOESM1_ESM.docx]

**Response to Reviewers' Comments**
Manuscript titled: "Mitral Valve Prosthesis Endocarditis Unveiling Metastatic Colorectal Cancer as the Primary Infection Source: A Case Study"

**1. Consent for Publication**
Thank you for highlighting this important point. We have now included a specific statement in the "Methods" section regarding consent for publication. Since this case involves data related to an individual patient, we obtained written consent for the publication of the case report. The added statement is as follows:

"Written informed consent for the publication of this case report was obtained from the patient prior to her death, in compliance with the journal’s guidelines."

If required, we are ready to provide a copy of the consent form at any time.

**2. Accurate Reporting of Results and Rewriting of Conclusions**
We have carefully reviewed the results section to ensure that all clinical details are reported accurately and in alignment with the actual clinical course. Specifically, we have clarified the progression under antibiotic treatment and the reduction of vegetation size. We have also made sure that the conclusions are appropriately tempered. Here is the revised version of the conclusions:

"This case highlights the importance of considering atypical sources, such as metastatic cancer, in unresolved cases of endocarditis. The complexity of managing concurrent cardiac infection and malignancy is evident and underscores the need for multidisciplinary collaboration. However, despite the initial favorable response to antibiotic therapy, the presence of an aggressive neoplasm significantly influenced the patient's overall prognosis."

**3. Full Explanation of Limitations**
We have expanded the discussion on limitations in the revised version. These include the absence of advanced therapeutic options for metastatic colorectal cancer due to the patient's premature death and the challenge of evaluating the long-term success of antibiotic therapy without extended follow-up. Below is the addition we made to the discussion:

"The main limitations of this case lie in the lack of long-term follow-up due to the patient’s premature death. This prevents a full assessment of the long-term effectiveness of the antibiotic therapy. Moreover, treatment options for metastatic colorectal cancer could not be explored, limiting the evaluation of potential interactions between oncologic treatments and the management of endocarditis."

**4. Submission of Revisions**
We have incorporated all the necessary changes into the revised manuscript and prepared a final version without track changes as required. Additionally, a version with track changes is included in the associated files to facilitate the verification of all modifications.

We hope that these revisions fully address the reviewers’ and editor’s expectations. Please feel free to contact us for any further clarifications or additional information.
