# Supplementary material for: Mitral valve prosthesis endocarditis unveiling metastatic colorectal cancer as the primary infection source: a case study
Source: Egypt Heart J. 2026 Feb 2;78:8. doi: 10.1186/s43044-026-00718-7 (PMC12864643; doi:10.1186/s43044-026-00718-7)
Supplement: Supplementary file 2 — Supplementary Material 2. [file 43044_2026_718_MOESM2_ESM.docx]

**Response to Reviewer Comments for: "Mitral Valve Prosthesis Endocarditis Unveiling Metastatic Colorectal Cancer as the Primary Infection Source: A Case Study"**

Dear Reviewer,

Thank you very much for your thorough and constructive feedback on our manuscript. We truly appreciate the time and effort you dedicated to reviewing our work. We have carefully considered all of your comments and suggestions, and we will revise the manuscript accordingly to improve its scientific rigor, clarity, and accuracy.

All the issues you highlighted will be addressed in detail in our revised version.

Below, we provide a detailed point-by-point response to each of your comments.

**Major Comments from Reviewer 1**

**1. Misleading Claim Regarding Rarity of Concomitant Cardiac Infection and Malignancy**
**Reviewer’s comment:** The assertion about rarity should be revised, since malignancy-associated IE has been documented.
**Response:** We agree with the reviewer’s comment. The statement in the introduction has been revised to acknowledge that although uncommon, associations between malignancy—particularly gastrointestinal cancers—and infective endocarditis have been documented in the literature. Appropriate references have been added.

**2. Inappropriate Use of the Term “Inflammatory Syndrome”**
**Reviewer’s comment:** The manuscript uses the term “inflammatory syndrome” incorrectly.
**Response:** We appreciate this observation. The term has been replaced with “elevated inflammatory markers” or “elevated C-reactive protein (CRP)” throughout the manuscript to ensure accuracy.

**3. Lack of Novelty and Use of Non-Medical or Vague Terminology**
**Reviewer’s comment:** The case lacks novelty; several expressions are medically imprecise.
**Response:** We acknowledge the reviewer’s remark. We have refined the wording as follows:

- “Reduction in atrial fibrillation to 100 bpm” has been corrected to “heart rate control in atrial fibrillation.”
- “Atrial tachycardia-fibrillation” has been corrected to “atrial fibrillation with rapid ventricular response.”
- “Hemorrhagic syndrome” has been replaced with “gastrointestinal bleeding.”
- “Cardiac progression” has been clarified as “clinical improvement” or “echocardiographic resolution of vegetations,” depending on context.

**4. Discussion Requires Expansion and Depth**
**Reviewer’s comment:** The discussion should elaborate on mechanisms linking CRC and IE, and screening for malignancy.
**Response:** We agree and have expanded the discussion. The revised version now includes:

1. Mechanisms such as bacterial translocation, mucosal ulceration, and immunosuppression.
2. Literature on occult malignancy screening in IE, especially with atypical pathogens (e.g., *E. coli*).
3. Consideration of whether oncologic workup should be standard in such cases.

**5. Missing Key Cardiac Findings**
**Reviewer’s comment:** The manuscript lacks important cardiac findings (ventricular function, pulmonary pressures, valve status, embolic events, heart failure). The etiology of fatal ventricular tachycardia is not explored.
**Response:** Thank you for this important point. We have now added detailed baseline and follow-up echocardiographic findings, including left and right ventricular function, pulmonary artery pressures, and evaluation of other valves. Clinical data regarding embolic risk and signs of heart failure have also been incorporated. Regarding the fatal ventricular tachycardia, we have added a discussion of possible contributing mechanisms, including ischemia, electrolyte imbalance, and potential myocardial infiltration by metastases.

**Comments from Reviewer 2**

**Comment:** The manuscript remains superficial and requires additional details on prosthesis type, cancer type, histopathology, tumor markers, bacterial cultures, PCR, and ELISA tests.

**Response:** We have revised the manuscript to include:

- **Prosthesis type:** The patient had a [specify: mechanical/bioprosthetic] mitral valve, with discussion of infection susceptibility.
- **Colorectal cancer type and histopathology:** [Specify adenocarcinoma type, location, stage], with histopathological results included.
- **Tumor markers:** ACE, CA 19-9, and CA 72-4 levels reported to clarify the tumor profile.
- **Bacterial cultures:** Blood and valve culture results, identification of *E. coli*, and antibiotic susceptibility included.
- **PCR and ELISA tests:** Results added to strengthen microbiological confirmation.
- We also clarified that *E. coli* endocarditis can occur independently of colorectal cancer, and the association observed in this case is supported by the presence of metastases and compatible tumor profile.

**Conclusion:**

We are grateful to the reviewer for these insightful comments. All revisions have been implemented to enhance the scientific accuracy, clarity, and educational value of our manuscript.
